# GPX4 Inhibition Enhances the Pro-Oxidant and ER Stress Effects of Tempol in Colon and Gastric Cancer Cell Lines

**DOI:** 10.3390/cimb47100856

**Published:** 2025-10-16

**Authors:** Gorkem Ozdemir, Halil Mahir Kaplan

**Affiliations:** 1 Department of Gastroenterological Surgery, Adana City Training and Research Hospital, 01230 Adana, Turkey; 2 Department of Pharmacology, Faculty of Medicine, Çukurova University, 01330 Adana, Turkey; mkaplan@cu.edu.tr

**Keywords:** ER stress, unfolded protein response, tempol, ML210, GPX4 inhibitor, IRE1α, ATF6, GRP78, gastric cancer, colon cancer

## Abstract

Tempol, a synthetic nitroxide, exhibits dual antioxidant and pro-oxidant activity, requiring millimolar concentrations to induce oxidative stress, which limits its therapeutic use. Glutathione Peroxidase 4 (GPX4) is a critical lipid peroxidase that prevents ferroptosis, and its inhibition has emerged as a strategy to sensitize cancer cells to oxidative stress. To enhance Tempol’s efficacy, we investigated its interaction with ML210, a GPX4 inhibitor, in human colon (HT29) and gastric (CRL-1739) cancer cell lines. We quantified cell viability, oxidative stress markers (H_2_O_2_, Total Oxidant Status (TOS), and Total Antioxidant Status (TAS)) and endoplasmic reticulum (ER) stress proteins (ATF6, GRP78, and IRE1α) in in vitro assays. Synergy was assessed using Bliss independence analysis. The combination of Tempol (2 mM) and ML210 (0.05 μM) markedly reduced viability in both cell lines. Bliss analysis revealed slight/moderate synergy for cytotoxicity (Δ = +0.15 in HT29; Δ = +0.26 in CRL-1739) and strong synergy for H_2_O_2_ accumulation (Δ = +1.92–2.23 across replicates). In contrast, TOS showed moderate-to-strong antagonism across both cell lines, and TAS demonstrated slight synergistic or antagonistic effects. ER stress markers exhibited marker and cell line specific synergy: ATF6 showed strong synergy, IRE1α slight synergy in both lines, and GRP78 activation was highly variable, showing strong synergy in CRL-1739 cells but moderate antagonism in HT29 cells. These findings indicate that the cooperative action of Tempol and ML210 is ROS-pool–specific and pathway-selective in the ER. These findings demonstrate that ML210 potentiates Tempol’s pro-oxidant pressure by targeting GPX4, selectively amplifying H_2_O_2_ accumulation and ER stress engagement without collapsing global redox balance. This study provides mechanistic rationale for redox–proteostasis co-targeting in gastric and colon cancers and establishes a foundation for in vivo validation.

## 1. Introduction

Gastrointestinal cancers, including gastric and colon carcinomas, represent a major global health burden, necessitating the development of novel and more effective therapeutic strategies [1]. Conventional chemotherapies remain limited by drug resistance and systemic toxicity, highlighting the importance of approaches that can improve efficacy while minimizing adverse effects [2]. Both reactive oxygen species (ROS) and endoplasmic reticulum (ER) stress play dual roles in cancer; while moderate levels may promote tumor survival, excessive induction can trigger cell death through oxidative damage and activation of the unfolded protein response (UPR) [3,4]. Consequently, modulating cellular redox homeostasis and ER function has therefore emerged as an anticancer therapy [2].

ER stress is strongly implicated in multiple cancers, including those of the digestive tract. The three primary ER stress sensors, PERK, IRE1, and ATF6, activate distinct signaling pathways upon separation from the ER chaperon glucose-regulated protein 78 (GRP78) [5]. These sensors function both independently and cooperatively. Their activation initially enhances protein-folding capacity to restore proteostasis, but when ER stress is prolonged or severe, the same pathways can induce apoptosis [6].

Tempol, a synthetic nitroxide, has gained attention for its diverse anticancer properties. It induces apoptosis and oxidative stress in several cancer models, including gastric and colon cell lines [7,8]. A key feature of Tempol is its dose-dependent functional inversion: at low concentrations, it acts as an antioxidant, whereas at millimolar levels, it switches to a pro-oxidant, generating ROS and driving cytotoxicity [9,10,11]. Although this dual functionality underpins its anticancer potential, the requirement for high concentrations raises concerns about clinical utility and toxicity.

Ferroptosis, an iron-dependent form of regulated cell death characterized by lipid peroxidation, has recently emerged as a vulnerability in aggressive and drug-resistant cancers [12]. Glutathione peroxidase 4 (GPX4) plays a central role in protecting cells from ferroptosis by reducing lipid hydroperoxides [13]. Unlike other glutathione peroxidases, GPX4’s distinct structure allows it to directly metabolize complex lipid peroxides [9,14,15]. Inhibitors of GPX4, such as ML210, effectively induce ferroptosis in diverse cancer models [16,17], including drug-resistant settings both in vitro and in vivo [18].

Given Tempol’s concentration-dependent redox switch and the critical role of GPX4 in restraining lipid peroxidation, we hypothesized that combining Tempol with ML210 would synergistically amplify oxidative and ER stress, thereby enhancing cytotoxicity at otherwise sub-cytotoxic doses. The present study was designed to evaluate the individual and combined effects of Tempol and ML210 on cell viability, oxidative stress, and ER stress markers in HT29 and CRL-1739 cell lines, providing mechanistic insight into their potential as a redox–proteostasis co-targeting strategy for gastrointestinal malignancies.

## 2. Materials and Methods

This study was carried out in the Department of Pharmacology, Faculty of Medicine, Çukurova University between the dates of 15 February 2025 and 15 June 2025.

### 2.1. Chemicals

Tempol, ML210, McCoy’s 5A Medium, RIPA buffer, fetal bovine serum, PBS, NaCl, TritonX-100, EGTA, dithiothreitol, NaF, Tris–HCl, Na3VO4 were acquired from Sigma-Aldrich, Inc. The Bradford dye reagent was purchased from Bio-Rad Laboratories, Inc. (Hercules, CA, USA).

### 2.2. Cell Culture

The human HT29 cell line was obtained from the American Type Culture Collection (ATCC, Manassas, VA, USA) and cultured in modified McCoy’s 5A medium supplemented with 10% fetal bovine serum (FBS, Gibco, Thermo Fisher Scientific, Waltham, MA, USA). The human gastric adenocarcinoma cell line CRL-1739 was maintained in F-12 medium (Gibco, Thermo Fisher Scientific, Waltham, MA, USA) supplemented with 100 μg/mL streptomycin, 100 U/mL penicillin (Sigma-Aldrich, St. Louis, MO, USA), and 10% FBS (Gibco, Thermo Fisher Scientific, Waltham, MA, USA). Both cell lines were incubated at 37 °C in a humidified atmosphere containing 5% CO_2_ and were routinely passaged every 4–5 days. Tempol and ML210 (Sigma-Aldrich, St. Louis, MO, USA) were dissolved in dimethyl sulfoxide (DMSO; Sigma-Aldrich, St. Louis, MO, USA). Control cells received fresh medium, while treatment groups were exposed to Tempol, ML210, or their combination for 48 h.

All experiments were performed using mycoplasma-negative cultures. Cells were used between passages 3–15, with CRL-1739 specifically limited to passages 5–12. Each condition was tested in at least three independent biological replicates with technical triplicates, and only replicate-confirmed data were included in statistical analyses.

### 2.3. Cell Viability and IC_50_ Determination (MTT Assay)

The effects of Tempol and ML210 on the proliferation of cultured cancer cells were determined using the MTT assay. Cells were seeded in 96-well plates at a density of 1 × 10^4^ cells per well. Following a 48-h treatment, we assessed viability, intracellular hydrogen peroxide (H_2_O_2_), total oxidative stress, total antioxidant status, and ER stress markers. For the combination experiments, we selected a fixed high dose of 2 mM Tempol together with 0.05 µM ML210.

Following treatment, cell viability was assessed using the MTT Cell Proliferation and Cytotoxicity Assay Kit (catalog no. E-CK-A341; Elabscience, Houston, TX, USA), according to the manufacturer’s instructions. Absorbance was measured at 570 nm with a microplate reader (EL800; BioTek Instruments, Inc., Winooski, VT, USA). Viability was expressed as a percentage relative to untreated control cells. Results were reported as mean ± standard deviation (SD).

### 2.4. Cell Homogenization

Following a 48 h treatment with Tempol, ML210, or their combination, cells were harvested and transferred to 15 mL conical tubes. Samples were centrifuged at 2000 rpm for 10 min at 4 °C. The supernatant was discarded, and the cell pellet was resuspended in 5 mL of phosphate-buffered saline (PBS). This wash step was repeated once under the same conditions. For protein extraction, the washed cells were lysed in 250 μL of RIPA buffer supplemented with protease inhibitors (2.5 μL PMSF [200 mM], 2.5 μL sodium vanadate [100 mM], and 2.5 μL protease inhibitor cocktail). Cell lysates were homogenized on ice using an ultrasonic homogenizer. Homogenates were centrifuged at 10,000 rpm for 10 min at 4 °C, and the supernatant was collected for subsequent analyses.

### 2.5. Total Protein Quantification

Total protein content was quantified using the Bradford assay. A standard curve was generated with bovine serum albumin (BSA) at concentrations of 1, 2, 3, 5, 7, 8, and 10 μg/mL. For each sample, 10 μL of homogenate was diluted to 100 μL with distilled water, followed by the addition of 1 mL of Bradford reagent. After thorough mixing, absorbance was measured at 595 nm with a spectrophotometer. All measurements were performed in six independent replicates. Protein concentrations (μg/μL) were determined by interpolation from the BSA standard curve, using GraphPad Prism version 10.5.0 (GraphPad Software, San Diego, CA, USA).

### 2.6. Quantification of Oxidative Stress Markers

#### 2.6.1. Intracellular Hydrogen Peroxide (H_2_O_2_) Level Quantification

Intracellular H_2_O_2_ levels were quantified using the ab102500 Hydrogen Peroxide Assay Kit (Abcam, plc, Cambridge, UK), following the manufacturer’s colorimetric protocol. Hydrogen Peroxide (H_2_O_2_) levels were quantified and compared.

#### 2.6.2. Total Antioxidant Status and Total Oxidant Status Measurement

Total oxidant status (TOS) and total antioxidant status (TAS) were quantified using commercial colorimetric assay kits (Rel Assay Diagnostics, Gaziantep, Turkey), in accordance with the manufacturer’s protocols. Absorbance was measured at 660 nm using a microplate reader, and results were expressed as mean ± standard deviation from independent replicates. The TAS levels were quantified and expressed as mmol Trolox equivalent per gram of protein (mmol Trolox Eq./g), while TOS levels were expressed as μmol H_2_O_2_ equivalent per gram of protein (μmol H_2_O_2_ Eq./g). All measurements were performed in six independent replicates.

### 2.7. Quantification of ER Stress Markers (ELISA)

Protein expression levels of ER stress markers were quantified by ELISA. Specifically, IRE1α (catalog no. NBP3–49699), GRP78 (catalog no. NBP2–62145), and ATF6 (catalog no. NBP2–69885) ELISA kits (Novus Biologicals, Centennial, CO, USA) were employed according to the manufacturer’s protocols. Each experiment was repeated six times independently.

### 2.8. Statistical Analysis

All statistical analyses were performed using GraphPad Prism version 10.5.0 (GraphPad Software, San Diego, CA, USA). Data are presented as mean ± standard deviation (SD).

Prior to hypothesis testing, normality of continuous variables (cell viability, oxidative stress markers, ER stress proteins) was evaluated using the Shapiro–Wilk test, and homogeneity of variances was assessed by Levene’s test. As no significant deviations were detected, parametric tests were applied. Between-group comparisons were performed using unpaired two-tailed Student’s *t*-tests or one-way ANOVA with appropriate post hoc analyses (Dunnett’s or Tukey’s). For transparency, data were also analyzed using the non-parametric Kruskal–Wallis test, which revealed comparable trends, thereby supporting the robustness of our conclusions.

Half-maximal inhibitory concentrations (IC_50_) were calculated by nonlinear regression (log[inhibitor] vs. normalized response, variable slope).

Drug–drug interactions were evaluated according to the Bliss independence model [19,20]. Fractional effects (E) were calculated by normalizing treatment responses to control means (E = 0 indicates no effect; E = 1 indicates complete effect). The Bliss-predicted effect of a two-drug combination (Eexp) was defined as:

Eexp = ET + EM − (ET × EM)Eexp = E_T + E_M − (E_T × E_M)Eexp = ET + EM − (ET × EM)

where ETE_TET and EME_MEM represent the individual effects of Tempol and ML210, respectively.

The observed effect (Eobs) was compared with Eexp to calculate the synergy score (Δ):

Δ = Eobs − Eexp Δ = Eobs − EexpΔ = Eobs − Eexp

Interactions were interpreted using predefined thresholds as follows: When the absolute synergy score (|Δ|) was equal to or greater than 0.50, the interaction was defined as strong synergy (if Δ > 0) or strong antagonism (if Δ < 0). Scores between 0.20 and 0.50 indicated a moderate interaction or antagonistic effect, while values between 0 and 0.20 reflected a slight synergistic or antagonistic effect. When Δ was approximately 0, the interaction was considered additive or independent.

## 3. Results

Mean protein concentrations and oxidative stress parameters for control, Tempol, ML210, and combination treatments are presented in Table 1, together with statistical comparisons (95% CI, *p*-values) demonstrating the enhanced effects of the combination.

### 3.1. Dose–Response and Single-Agent Activity

**Figure 1:**
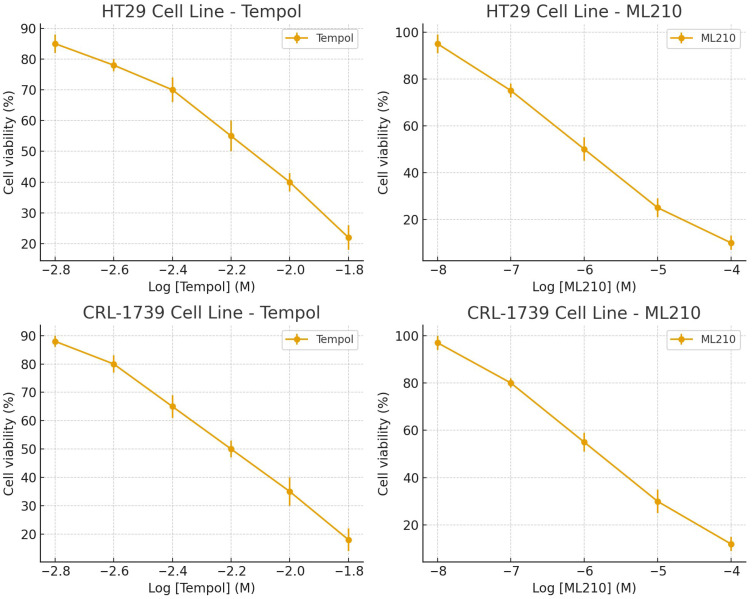
Dose–response curves for Tempol and ML210 in HT29 and CRL-1739 cell lines. Cell viability (%) was measured across a range of concentrations and expressed as mean ± SEM. The curves were used to estimate half-maximal inhibitory concentrations (IC_50_ values). (Raw values: for Tempol, in mM; for ML210, in µM.).

The half-maximal inhibitory concentration (IC_50_) values for Tempol and ML210 in CRL-1739 and HT29 cells are shown in Table 2. (Figure 1) Tempol and ML210 reduced viability in a dose-dependent manner.

### 3.2. Combination Cytotoxicity and Bliss Independence Analysis (Cell Viability)

**Figure 2:**
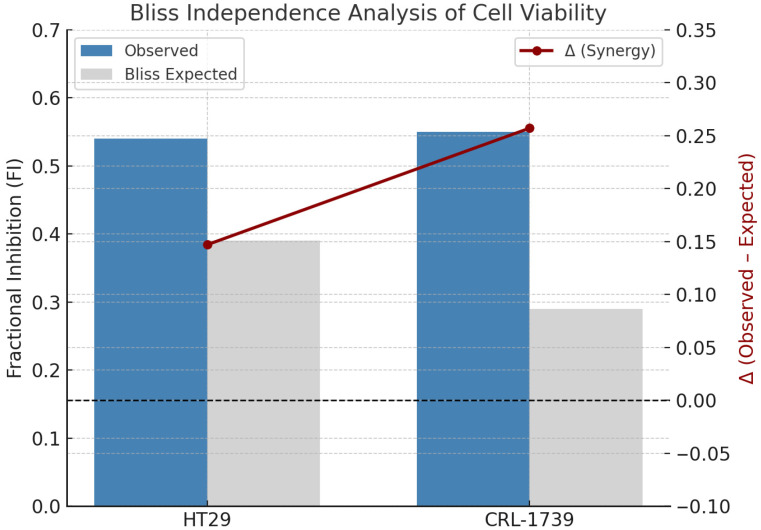
Bliss independence analysis of cell viability in CRL-1739 and HT29 cells. Bars represent observed vs. Bliss-expected fractional inhibition (FI, mean ± SEM). The red line shows Δ values (Observed–Expected), demonstrating slight but significant synergy in HT29 (Δ = +0.147 ± 0.013, *p* < 0.05) and moderate synergy in CRL-1739 (Δ = +0.257 ± 0.038, *p* < 0.05) (see Table 4). Y-axes: Fractional Inhibition (FI) (left) and Δ (Observed–Expected) (right).

At the combination doses tested, cell viability was reduced by nearly 50% relative to controls, which corresponds to an approximate two-fold greater inhibition than achieved with either single agent (Table 3). Bliss analysis revealed (Table 4, Figure 2) slight-to-moderate synergy in both lines.

### 3.3. Oxidative Stress: H_2_O_2_, TOS, TAS Indices

**Figure 3:**
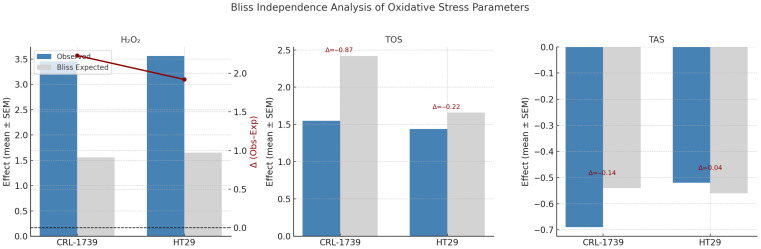
Bliss independence analysis of oxidative stress parameters in CRL-1739 and HT29 cells. Bars show observed vs. Bliss-expected effects (mean ± SEM) for H_2_O_2_, TOS, and TAS. The red line indicates Δ values (Observed–Expected). Statistical significance of Δ was assessed by one-sample *t*-tests (see Table 5). H_2_O_2_ exhibited strong synergy in both lines, while TOS showed antagonism and TAS minimal effects. The horizontal dotted line in the H_2_O_2_ plot denotes the zero-effect (Δ = 0) reference level, representing the Bliss-expected additive response. Y-axes: Effect (mean ± SEM) (left) and Δ (Observed–Expected) (right).

Combination treatment selectively amplified intracellular H_2_O_2_ accumulation, producing >2-fold higher levels than expected from additivity (Table 5, Figure 3). This contrasted with global oxidant and antioxidant indices:

TOS increased under both single agents and combination but the combined effect was antagonistic.

TAS decreased across treatments, but combination effects did not exceed Bliss expectations, showing only slight synergistic or antagonistic effects.

Thus, synergy was ROS-pool specific, strongest for H_2_O_2_ rather than for generalized oxidative burden.

### 3.4. ER Stress Markers

**Figure 4:**
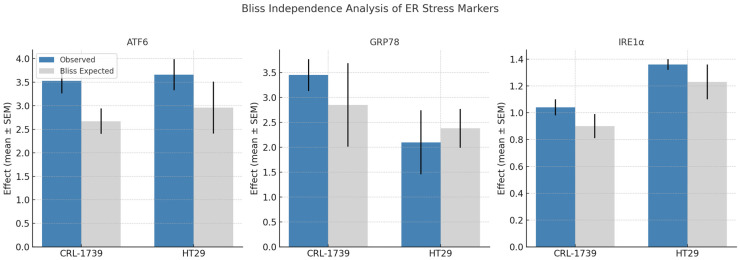
Data are presented as observed vs. Bliss-expected normalized protein concentrations (mean ± SEM). Statistical significance of Δ values was assessed by one-sample *t*-tests (see Table 6). ATF6 showed strong synergy in both cell lines. IRE1α exhibited slight, non-significant synergy in both CRL-1739 and HT29. GRP78 responses were cell line–dependent, with strong synergy in CRL-1739 but no significant difference (moderate antagonism) in HT29 (*p* ≥ 0.05).

Combination treatment enhanced ER stress signaling with varying degrees of synergy (Table 6, Figure 4).

ATF6 showed strong synergy, with combination effects ~2–3-fold above single agents, whereas IRE1α showed slight synergy in both cell lines.

GRP78 activation shows a cell-line specific synergy pattern when Tempol and ML210 are combined, resulting in strong synergy in CRL-1739 cells (3.45-fold activation, Δ = +0.94) but moderate antagonism in HT29 cells (2.10-fold activation, Δ = −0.29).

These findings indicate that GPX4 inhibition potentiates Tempol’s pro-oxidant activity in a way that couples specifically to ER stress activation, especially via ATF6.

### 3.5. Integrated Summary

Combination of Tempol and ML210 displayed a pattern of selective synergy:

Cytotoxicity and H_2_O_2_ generation were consistently synergistic.

ER stress markers showed synergy of varying strength, with ATF6 most responsive for both cell lines.

Global redox indices (TOS, TAS) showed synergistic or antagonistic outcomes, underscoring that synergy was not uniform across oxidative pathways.

Together, these results support a mechanistic model where GPX4 inhibition amplifies Tempol’s pro-oxidant activity, producing targeted oxidative stress and ER stress responses that converge on cytotoxicity.

## 4. Discussion

Our data demonstrate that Tempol, when combined with the GPX4 inhibitor ML210, exerts selective and pathway-specific synergy in gastric and colon cancer cells. Bliss independence analysis revealed three consistent patterns: (i) strong synergy for H_2_O_2_ accumulation in both cell lines; (ii) marker-dependent synergy in ER stress signaling, strongest for ATF6, and (iii) absence of synergy for global redox indices—TOS and TAS. These findings indicate that the cooperative action is driven by specific ROS pools and ER-targeted effects rather than a generalized collapse of redox homeostasis.

The current study employed a 2 mM Tempol dose, based on our prior work in HT29 and CRL-1739 cells, which showed that this dose at 48 h triggers strong pro-apoptotic signaling (e.g., Bax, cleaved caspase-3) and endoplasmic reticulum (ER) stress markers [7]. However, this strong signaling activation at 48 h only indicates the initiation of the apoptotic program, not its completion. The current manuscript confirms that at 48 h, 2 mM Tempol results in limited functional cytotoxicity without causing widespread cell death (~80–85% survival). This means that while the cell is highly stressed and actively signaling for apoptosis (consistent with the previous paper’s findings), the majority of the cells have not yet undergone membrane collapse or metabolic failure as measured by the MTT assay. Importantly, the previous study did not quantify cell viability; it focused instead on molecular markers of apoptosis and stress. The 2 mM dose was chosen as a sub-maximal probe to detect synergistic interactions with GPX4 inhibition, which would be obscured at fully cytotoxic levels. Thus, the two studies complement each other: the former defined Tempol’s intrinsic pro-oxidant activity, while the current work demonstrates how GPX4 inhibition enhances these effects within oxidative and ER stress pathways.

This prominent H_2_O_2_-specific synergy aligns with the dual nature of Tempol. At millimolar concentrations, Tempol shifts from antioxidant to pro-oxidant activity, producing sustained ROS [7,8,9,15]. In parallel, ML210 reduces the cell’s capacity to detoxify lipid peroxides, thereby amplifying Tempol’s pro-oxidant pressure [13,16,17]. The observation that synergy is confined to H_2_O_2_, but not to TOS or TAS, suggests that localized ROS species and microdomains—rather than overall oxidant load—drive the cooperative effect. Oxidative damage is therefore sufficiently targeted to activate ER stress signaling without depleting total antioxidant reserves.

ER stress markers further support this model. ATF6 showed strong synergy, IRE1α showed slight synergy, and GRP78 showed variable responses between cell lines. These results confirm previous reports that ROS, including H_2_O_2_ and lipid peroxides, can disrupt ER proteostasis and activate the UPR [1,9,19,20,21]. Inhibition of GPX4 is mechanistically linked to lipid peroxidation and ferroptosis [22,23,24,25,26,27,28,29], and increasing evidence suggests crosstalk between ferroptosis and ER stress [25,29,30]. This framework is consistent with studies identifying GPX4 as a gatekeeper of lipid peroxide toxicity and ferroptosis [12,15,16,22,23,24,25,26] and with reports that excess H_2_O_2_ is cytotoxic and capable of triggering apoptosis or other cell-death programs when antioxidant buffering is exceeded [27,28,29,30,31,32]. The ER stress response exhibited a selective pattern, with ATF6 showing the strongest synergy, while GRP78 was variable, and IRE1α showed slight synergy. This hierarchy is consistent with literature indicating that oxidative and lipid peroxidation stress preferentially engage ATF6, which can drive antioxidant programs such as catalase induction, thereby linking ROS and ER signaling. GRP78, as a general chaperone, is more variable and highly context-dependent, while IRE1α activation is often modest or transient in oxidative settings and has been linked to ferroptosis sensitivity. Thus, the ATF6-biased response observed here aligns with mechanistic precedents reported in redox–proteostasis literature [33,34].

Several limitations should be noted. Mechanistic insight would be enhanced by direct measurement of lipid peroxidation, ferroptosis markers, and dynamic UPR activation. The current work relied on in vitro assays with relatively small sample sizes, and the precise mode of cell death (apoptosis vs. ferroptosis) was not definitively established. Although GPX4 inhibition is mechanistically linked to ferroptosis, our study did not include direct ferroptosis markers such as lipid peroxidation, glutathione depletion, or pharmacological rescue assays. Consequently, our findings should be interpreted as demonstrating synergy consistent with ferroptosis-associated mechanisms, but not as definitive proof of ferroptosis induction. Future work incorporating direct ferroptosis assays will be essential to validate this pathway. The present study was limited to one gastric (CRL-1739) and one colorectal (HT29) cancer cell line. These were selected as representative models of gastrointestinal malignancies that share overlapping biology and therapeutic challenges. While this design allowed us to test for conserved mechanistic interactions between Tempol and GPX4 inhibition, it does not exclude the possibility of cell line–specific responses. Future studies incorporating multiple independent lines from each tumor type, and ideally patient-derived models, will be essential to confirm the generalizability of our findings.

From a clinical perspective, Tempol’s systemic safety profile remains largely undefined; existing human data are limited to topical applications with mild adverse events [35]. The possibility that synergy with GPX4 inhibition could reduce effective dosing is an attractive therapeutic strategy. However, extensive preclinical testing will be essential to rule out off-target redox effects and establish systemic tolerability in vivo.

## 5. Conclusions

The combination of Tempol and ML210 yields a distinct synergy signature: potentiation of H_2_O_2_-driven oxidative stress, selective activation of ER stress pathways (ATF6 > IRE1α, GRP78 cell-type dependent), and lack of effect on global redox indices. This supports a model in which GPX4 inhibition amplifies Tempol’s pro-oxidant activity within discrete ROS pools, thereby inducing ER stress and cytotoxicity without collapsing overall redox balance. Together, these findings provide a rationale for redox–proteostasis co-targeting in colon and gastric cancer and establish the basis for subsequent in vivo validation.

## Figures and Tables

**Table 1:** Effect of Tempol and TPL + ML210 Combination on Protein Concentration in Cancer Cell Lines. (Bliss analyses–normalized values).

ELISA-Based Protein Concentrations	Cell Line	Control Mean ± SD	Tempol Alone [Tempol (2 mM)] Mean ± SD	ML210	Combination Treatment [Tempol (2 mM) + ML210 (0.05 μM)] (Mean ± SD)	*p*-Value *, 95%CI of Diff (Lower to Upper)	*p*-Value **, 95%CI of Diff (Lower to Upper)
IRE1α (pg/mL)	CRL-1739	110.00 ± 6.96	144.2 ± 9.99	20.5 ± 2.36	224.2 ± 17.44	0.0004, −51.46 to −16.87	<0.0001, −131.5 to −96.87
	HT29	100.8 ± 8.28	132 ± 10.86	19 ± 2.36	238 ± 12	0.0002, −45.95 to −16.38	<0.0001, −152.0 to −122.4
ATF6 (pg/mL)	CRL-1739	0.22 ± 0.05	0.36 ± 0.08	0.48 ± 0.16	0.98 ± 0.14	0.0549, −0.28 to 0.003	<0.0001, −0.91 to −0.62
	HT29	0.21 ± 0.08	0.42 ± 0.09	0.44 ± 0.21	0.99 ± 0.16	0.0147, −0.37 to −0.04	<0.0001, −0.94 to −0.61
GRP78 (pg/mL)	CRL-1739	0.35 ± 0.06	0.68 ± 0.15	0.78 ± 0.37	1.57 ± 0.28	0.0171, −0.59 to −0.06	<0.0001, −1.48 to −0.95
	HT29	0.43 ± 0.10	0.79 ± 0.10	0.92 ± 0.25	1.34 ± 0.63	0.2109, −0.88 to 0.18	0.0015, −1.44 to −0.38
H_2_O_2_ level μM/10^6^ Cell	CRL-1739	6.23 ± 0.38	6.62 ± 1.02	13.66 ± 3.07	28.50 ± 3.01	0.9113, −2.99 to 2.23	<0.0001, −24.87 to −19.66
	HT29	4.68 ± 0.40	4.32 ± 0.62	13.17 ± 3.06	21.33 ± 2.58	0.8862, −1.81 to 2.55	<0.0001, −18.83 to −14.47
TOS (μmol H_2_O_2_ Eq./g)	CRL-1739	9.67 ± 1.2	15.17 ± 1.32	21 ± 2.36	24.67 ± 3.2	0.0012, −8.68 to −2.31	<0.0001, −18.18 to −11.82
	HT29	9.50 ± 1.04	13.67 ± 1.97	19 ± 2.36	23.17 ± 3.43	0.0206, −7.71 to −0.62	<0.0001, −17.21 to −10.12
TAS (mmol Trolox Eq./g)	CRL-1739	1.85 ± 0.10	1.38 ± 0.15	1.19 ± 0.12	0.58 ± 0.20	0.0003, 0.23 to 0.70	<0.0001, 1.03 to 1.50
	HT29	1.87 ± 0.15	1.37 ± 0.20	1.19 ± 0.13	0.90 ± 0.25	0.0017, 0.20 to 0.80	<0.0001, 0.66 to 1.27

Data are presented as mean ± SD. Comparisons were performed using unpaired two-tailed Student’s *t*-tests (control vs. Tempol, control vs. combination). 95% confidence intervals of the difference and *p*-values are reported. *p* *: control vs. Tempol alone, *p* **: control vs. combined treatment. Abbreviations: SD, standard deviation; CI, confidence interval; TOS, Total Oxidant Status; TAS, Total Antioxidant Status; H_2_O_2_, hydrogen peroxide.

**Table 2:** IC_50_ values of Tempol and ML210 in gastric (CRL-1739) and colon (HT29) cancer cells.

Cell Line	Tempol IC_50_ (mM, Mean ± SD)	ML210 IC_50_ (µM, Mean ± SD)
CRL-1739	3.44 ± 0.50	0.49 ± 0.10
HT29	3.75 ± 0.30	0.44 ± 0.15

Data are presented as IC_50_ values (mean ± SD). IC_50_ values were calculated by nonlinear regression (log[inhibitor] vs. normalized response, variable slope). Abbreviations: IC_50_, half-maximal inhibitory concentration; SD, standard deviation.

**Table 3:** Cell viability after Tempol, ML210, and combination treatment.

Cell Line	Tempol (2 mM)	ML210 (0.05 µM)	Combination	Statistical Comparison
CRL-1739	81.14 ± 4.87%	85.71 ± 3.49%	44.28 ± 7.06%	Combo vs. Tempol: *p* < 0.0001; Combo vs. ML210: *p* < 0.0001
HT29	84.42 ± 3.78%	85.00 ± 3.05%	41.28 ± 4.95%	Combo vs. Tempol: *p* < 0.0001; Combo vs. ML210: *p* < 0.0001

Data are presented as % viability (mean ± SD). Group comparisons (combination vs. monotherapies) were analyzed using one-way ANOVA with Tukey’s post hoc test. Abbreviations: SD, standard deviation; ANOVA, analysis of variance.

**Table 4:** Bliss independence analysis of cell viability.

Cell Line	Δ (Mean ± SEM)	Interpretation
HT29	+0.147 ± 0.013	Slight synergy
CRL-1739	+0.257 ± 0.038	Moderate synergy

Data are presented as Δ ± SEM. Δ represents the difference between observed and Bliss-expected fractional inhibition (Δ = Eobs − Eexp). Bliss synergy significance was tested by one-sample *t*-test against zero. Abbreviations: Δ, synergy index; SEM, standard error of the mean.

**Table 5:** Bliss independence analysis of oxidative stress parameters.

Parameter	Cell Line	Observed Effect (Mean ± SEM)	Bliss Expected	Δ (Obs–Exp)	Interpretation
H_2_O_2_	CRL-1739	3.45 ± 0.16	1.56 ± 0.25	+2.23 ± 0.36	Strong synergy
	HT29	3.56 ± 0.20	1.65 ± 0.26	+1.92 ± 0.16	Strong synergy
TOS	CRL-1739	1.55 ± 0.14	2.42 ± 0.25	−0.87 ± 0.18	Strong Antagonism
	HT29	1.44 ± 0.14	1.66 ± 0.18	−0.22 ± 0.32	Moderate antagonism
TAS	CRL-1739	−0.69 ± 0.05	−0.54 ± 0.03	−0.14 ± 0.06	Slight/antagonism
	HT29	−0.52 ± 0.05	−0.56 ± 0.04	+0.04 ± 0.03	Slight synergy

Data are presented as observed effect, Bliss-expected effect, and Δ (mean ± SEM). Bliss independence analysis was used to determine synergy, additivity, or antagonism. Δ values were tested against zero with one-sample *t*-tests. Abbreviations: H_2_O_2_, hydrogen peroxide; TOS, Total Oxidant Status; TAS, Total Antioxidant Status; Δ, synergy index; SEM, standard error of the mean.

**Table 6:** Bliss independence analysis of ER stress markers (ATF6, GRP78, IRE1α).

Marker	Cell Line	Observed Effect (Mean ± SEM)	Bliss Expected	Δ (Obs–Exp)	Interpretation
ATF6	CRL-1739	3.53 ± 0.27	2.67 ± 0.27	+0.86 ± 0.12	Strong synergy
	HT29	3.66 ± 0.33	2.96 ± 0.55	+1.38 ± 0.74	Strong synergy
GRP78	CRL-1739	3.45 ± 0.32	2.85 ± 0.84	+0.94 ± 1.01	Strong synergy
	HT29	2.10 ± 0.64	2.38 ± 0.39	−0.29 ± 0.69	Moderate antagonism
IRE1α	CRL-1739	1.04 ± 0.06	0.90 ± 0.09	+0.13 ± 0.07	Slight synergy
	HT29	1.36 ± 0.04	1.23 ± 0.13	+0.13 ± 0.12	Slight synergy

Data are presented as observed effect, Bliss-expected effect, and Δ (mean ± SEM). Bliss independence analysis was applied for ER stress markers (ATF6, GRP78, IRE1α). Statistical significance of Δ values was assessed by one-sample *t*-tests. Abbreviations: ATF6, Activating Transcription Factor 6; GRP78, Glucose-Regulated Protein 78; IRE1α, Inositol-Requiring Enzyme 1α; Δ, synergy index; SEM, standard error of the mean.

## Data Availability

The data that support the findings of this study are available from the corresponding author upon reasonable request. The human HT29 cell line was obtained from the American Type Culture Collection (ATCC).

## Abbreviations

ATF6	Activating Transcription Factor 6
BSA	Bovine Serum Albumin
CHOP	C/EBP Homologous Protein
CO2	Carbon Dioxide
CRL	Cell Repository Line
DMEM	Dulbecco’s Modified Eagle Medium
DMSO	Dimethyl Sulfoxide
EGTA	Ethylene glycol-bis(β-aminoethyl ether)-N,N,N′,N′-tetraacetic acid
ELISA	Enzyme-Linked Immunosorbent Assay
ER	Endoplasmic Reticulum
FBS	Fetal Bovine Serum
FDA	Food and Drug Administration
GPX4	Glutathione Peroxidase 4
GRP78	Glucose-Regulated Protein 78
H2O2	Hydrogen Peroxide
IC_50_	Half Maximal Inhibitory Concentration
IRE1α	Inositol-Requiring Enzyme 1 alpha
JNK	c-Jun N-terminal Kinase
mM	millimolar
MTT	3-(4,5-dimethylthiazol-2-yl)-2,5-diphenyltetrazolium bromide
NaCl	Sodium chloride
NaF	Sodium fluoride
nm	Nanometer
OD	Optical Density
PBS	Phosphate-Buffered Saline
PERK	Protein Kinase R-like ER Kinase
RIPA	Radioimmunoprecipitation Assay
ROS	Reactive Oxygen Species
rpm	Revolutions Per Minute
SD	Standard Deviation
Tris-HCl	Tris hydrochloride
UPR	Unfolded Protein Response
μg	Microgram
μL	Microliter
μM	Micromolar
